# Supplementary material for: Which Surrogate Marker of Insulin Resistance Among Those Proposed in the Literature Better Predicts the Presence of Non-Metastatic Bladder Cancer?
Source: J Clin Med. 2025 Apr 11;14(8):2636. doi: 10.3390/jcm14082636 (PMC12027915; doi:10.3390/jcm14082636)
Supplement: Supplementary file 1 [file jcm-14-02636-s001.zip › jcm-3542899-supplementary.pdf]

Supplementary Files

Table S1. Histology of the bladder cancer patients

Grading	Freq.	Percent	CUM.
0	3	2.44	2.44
1	3	2.44	4.88
3	4	4	8.13
G1	38	30.89	39.02
G3	75	60.98	100.00
Total	123	100	

Staging	Freq.	Percent	CUM.
0	1	0.81	0.81
pt1 N0 M0	1	0.81	1.63
pT1	39	31.71	33.33
pT1 N0	1	0.81	34.15
pT2a	16	13.01	47.15
pT2b	1	0.81	47.97
pT3 N0 Mx	1	0.81	48.78
pT3a N0 Mx	2	1.63	50.41
pT4a Nx Mx	1	0.81	51.22
pTa	58	47.15	98.37
pTis	2	1.63	100
Total	123	100	
